# Clinical Usefulness of the MINI (Mucosal Inflammation Noninvasive Index) Score as a Non-Invasive Indicator for Assessing Mucosal Healing in Pediatric Patients with Crohn’s Disease

**DOI:** 10.3390/jcm14238331

**Published:** 2025-11-24

**Authors:** Katarzyna Akutko, Tomasz Pytrus

**Affiliations:** 2nd Clinical Department of Paediatrics, Gastroenterology and Nutrition, Faculty of Medicine, Wroclaw Medical University, 50-367 Wroclaw, Poland; tpytrus@usk.wroc.pl

**Keywords:** Mucosal Inflammation Noninvasive Index (MINI), Crohn’s disease, children

## Abstract

**Background/Objectives:** The primary goal of Crohn’s disease (CD) treatment is to achieve mucosal healing (MH). The best test to assess the effectiveness of therapy is endoscopic examination of the gastrointestinal (GI) tract, which is an invasive examination associated with a potentially high risk of complications resulting from the examination technique itself and the general anesthesia procedure. Previously used noninvasive methods for assessing the activity of CD show an unsatisfactory correlation with the severity of endoscopic lesions. There is a need to develop new, noninvasive indicators of the severity of inflammatory lesions in the GI tract in the course of CD. The aim of the study was to assess the clinical usefulness of the Mucosal Inflammation Noninvasive Index (MINI) as a noninvasive indicator of MH in pediatric patients with CD. **Methods**: The study included 199 children with CD who underwent endoscopic examinations of the upper and lower GI tract. The study group consisted of both patients with a diagnosis of CD and those whose indication for esophagogastroduodenoscopy and colonoscopy was a suspicion of inflammatory bowel disease. The clinical activity of CD was assessed using the Pediatric Crohn’s Disease Clinical Activity Index—PCDAI, and the severity of endoscopic inflammatory lesions was assessed using the simplified scale of endoscopic Crohn’s disease activity—SES-CD. The study assessed the correlation between the results of laboratory tests, PCDAI, SES-CD, and MINI. **Results**: In the study group positive correlation was found between MINI and PCDAI (r = 0.52, *p* < 0.001) and between MINI and SES-CD (r = 0.54, *p* < 0.001). A MINI score of 15 points or more indicated severe CD, defined as SES-CD ≥ 16 points, with a diagnostic sensitivity of 86% and specificity of 89%. Additionally, MINI was shown to be positively correlated with white blood cell count (WBC; r = 0.39, *p* < 0.001) and platelet count (PLT; r = 0.59, *p* = 0.007). **Conclusions**: MINI shows moderate correlation with endoscopic and clinical indices and may serve as a complementary, non-invasive marker for the assessment of MH in pediatric CD. Additional advantages of MINI are non-invasiveness, objectivity, and simplicity.

## 1. Introduction

In recent decades, a dynamic evolution of the treatment goals for Crohn’s disease (CD) in children has been observed. Achieving clinical remission alone (i.e., reducing the severity of clinical symptoms) has proven insufficient to alter the natural course of the disease and minimize the risk of complications. Numerous studies have shown that the primary treatment goal in children is to achieve mucosal healing (MH), which significantly improves the prognosis in long-term follow-up [1,2,3], including prolonged clinical remission, reduced number of hospitalizations, need for surgical intervention, and improved quality of life [4,5,6,7]. Serum and fecal biomarkers and clinical activity scales used in everyday clinical practice to assess the effectiveness of therapy in achieving MH have proven to be insufficiently sensitive and specific in terms of the specific intensity of inflammatory lesions in the intestines. The gold standard for monitoring the effectiveness of CD treatment in children remains endoscopic examination of the gastrointestinal (GI) tract [3,4]. Performing a colonoscopy only to assess the effectiveness of therapy is not recommended because it is an invasive procedure, the performance of which in pediatric patients is associated with the need for hospitalization and general anesthesia. It is also necessary to cleanse the large intestine, which is often poorly tolerated by children. Moreover, endoscopic procedures are expensive and constitute a significant burden for public health care [4,5,6].

Currently, the Pediatric Crohn’s Disease Activity Index (PCDAI) is widely used to assess the clinical activity of CD in children, and the Simple Endoscopic Crohn’s Disease Index (SES-CD) is used to assess endoscopic activity. PCDAI is a pediatric scale that considers objective aspects relevant to this age group and directly related to the clinical activity of CD (developmental parameters), as well as laboratory test results (albumin level, erythrocyte sedimentation rate—ESR). It also considers non-objective aspects related to functional limitations in daily life (general well-being, limitations in physical activity). PCDAI is particularly useful in assessing changes in CD activity over short periods and identifying patients with exacerbations. However, PCDAI has certain limitations. Most notably, it has a weak correlation with endoscopic disease activity, making the PCDAI less useful as a marker of persistent inflammation in asymptomatic children. SES-CD is used directly to assess the severity of inflammatory changes in individual sections of the intestine. A significant limitation of this scale is the requirement for endoscopic examination of the lower GI tract. This limits the usefulness of the SES-CD in everyday clinical practice. Furthermore, previous studies have shown insufficient correlation between MH and isolated biomarkers, i.e., C-reactive protein (CRP), ESR, fecal calprotectin (FC), and PCDAI. Therefore, there is a need to replace invasive, expensive methods of monitoring CD treatment in children with new, objective, non-invasive, inexpensive, and reproducible methods that do not burden patients in assessing the severity of inflammation in the GI tract [3,5]. It has been suggested that the simultaneous interpretation of clinical symptoms and non-invasive biomarkers may correlate better with SES-CD [8]. A scale combining various non-invasive markers, including FC levels, was needed, which could be an alternative to endoscopy in the assessment of MH [4,9].

Cozijnsen et al. [6] developed a new non-invasive index of mucosal inflammation that assesses the following components: stool consistency, FC, ESR, and CRP. Based on data from the multicenter, prospective ImageKids study, the associations between individual components of the PCDAI, laboratory parameters, and the SES-CDs were analyzed, and then a categorized index called the Mucosal Inflammation Noninvasive Index (MINI) was created. MINI correlates with SES-CD and may be useful in differentiating MH from mucosal content in children with CD. MINI was then validated in three independent patient cohorts. Cozijnsen et al. demonstrated that the combined interpretation of clinical symptoms and various laboratory parameters allows for a better assessment of MH than single biomarkers, including FC in children with CD [6,10].

The MINI is calculated based on clinical data that are routinely assessed and measured in all CD patients at every stage of the diagnostic and therapeutic process. This allows for better use of available data to reduce the need for invasive diagnostic and follow-up tests in everyday clinical practice, which may have a real impact on improving the quality of life of children with CD [5,6].

## 2. Aim

The aim of this study was to analyze the clinical usefulness of using MINI as a non-invasive indicator of MH assessment in pediatric patients with CD.

## 3. Materials and Methods

The results of endoscopic examinations of the upper and lower GI tract and medical records of children with CD hospitalized in the 2nd Clinical Department of Pediatrics, Gastroenterology, and Nutrition, Faculty of Medicine, Medical University of Wrocław between 2018 and 2023 were retrospectively analyzed. These examinations were performed both to establish the diagnosis and in children already diagnosed with CD. After initial analysis, patients who did not undergo a complete colonoscopy and/or gastroscopy and those in whom all parameters necessary for calculating the PCDAI and MINI were not assessed were excluded. Ultimately, data from 199 pediatric patients with CD were included in the study. The location of inflammatory lesions and the CD phenotype were described according to the Paris classification. Data on stool quantity and consistency were collected from patients’ medical records. Liquid stools were defined as those corresponding to type 7 on the Bristol Stool Chart, and semi-liquid stools were defined as those corresponding to type 6. Both PCDAI and MINI were assessed based on parameters obtained upon admission. According to PCDAI, clinical remission was defined as PCDAI < 10 points, mild disease as 11–25 points, moderate disease as 26–50 points, and severe disease as >50 points [11,12]. The severity of endoscopic inflammatory lesions was assessed using SES-CD, which is presented in Table 1 [13]. Depending on the total number of points, endoscopic CD activity was classified as no activity, 0–2 points; mild form, 3–6 points; moderate form, 7–15 points; severe form, ≥16 points. To calculate MINI, it is necessary to assess stool consistency in the previous week, FC, ESR, and CRP. The minimum MINI value is 3 points and the maximum is 25 points (Table 2) [6].

Medical records of patients qualified for the study were analyzed. Data on anthropometric measurements, course of CD, treatment used, location, and intensity of inflammatory lesions in the GI tract were taken into account. In addition, blood test results were analyzed (WBC, PLT, HGB) and inflammatory markers (CRP, ESR). The correlation between MINI and laboratory test results, PCDAI, and SES-CD was assessed.

### Statistical Analysis

Statistical analysis was performed using Statistica 13.3 PL (StatSoft Inc., Tulsa, OK, USA). Descriptive statistics measures were calculated for the studied features: mean, standard deviation (SD), and median. Normality of variables’ distribution was assessed using the Kolmogorov–Smirnov test. Data between groups were compared using the Student *t*-test (normally distributed quantitative variables) or the Mann–Whitney test (non-normally distributed quantitative variables). Spearman’s correlation was run to determine the relationship between MINI and PCDAI, SES-CD. ROC curve analysis was used to assess the diagnostic accuracy of MINI score to detect severe CD defined as SES-CD ≥ 16 points. The cut-off point was selected at the maximum Youden index. A *p*-value < 0.05 was considered statistically significant.

## 4. Results

In the study group, 28.14% were patients with MH defined as 0–2 points according to SES-CD, and 71.86% with active inflammatory lesions. The detailed characteristics of the study group are presented in Table 3.

**Figure 1:**
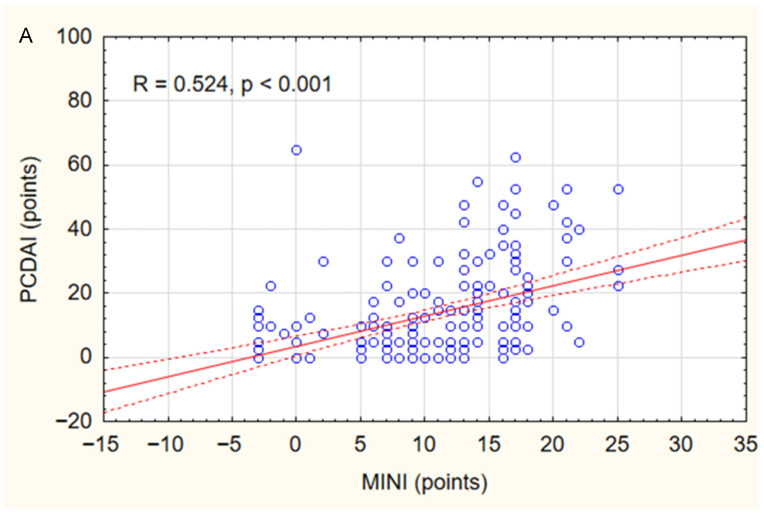

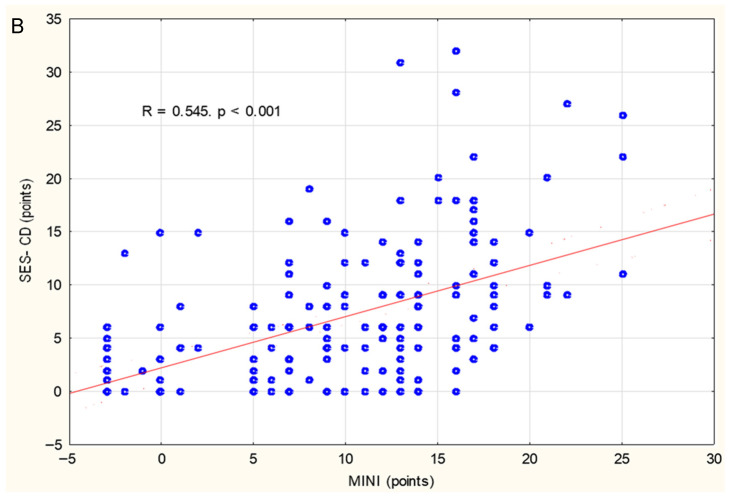
Correlations between MINI and PCDAI (**A**) and SES-CD (**B**) in the study group. Spearman correlation coefficients (R) and *p* values are reported.

**Figure 2:**
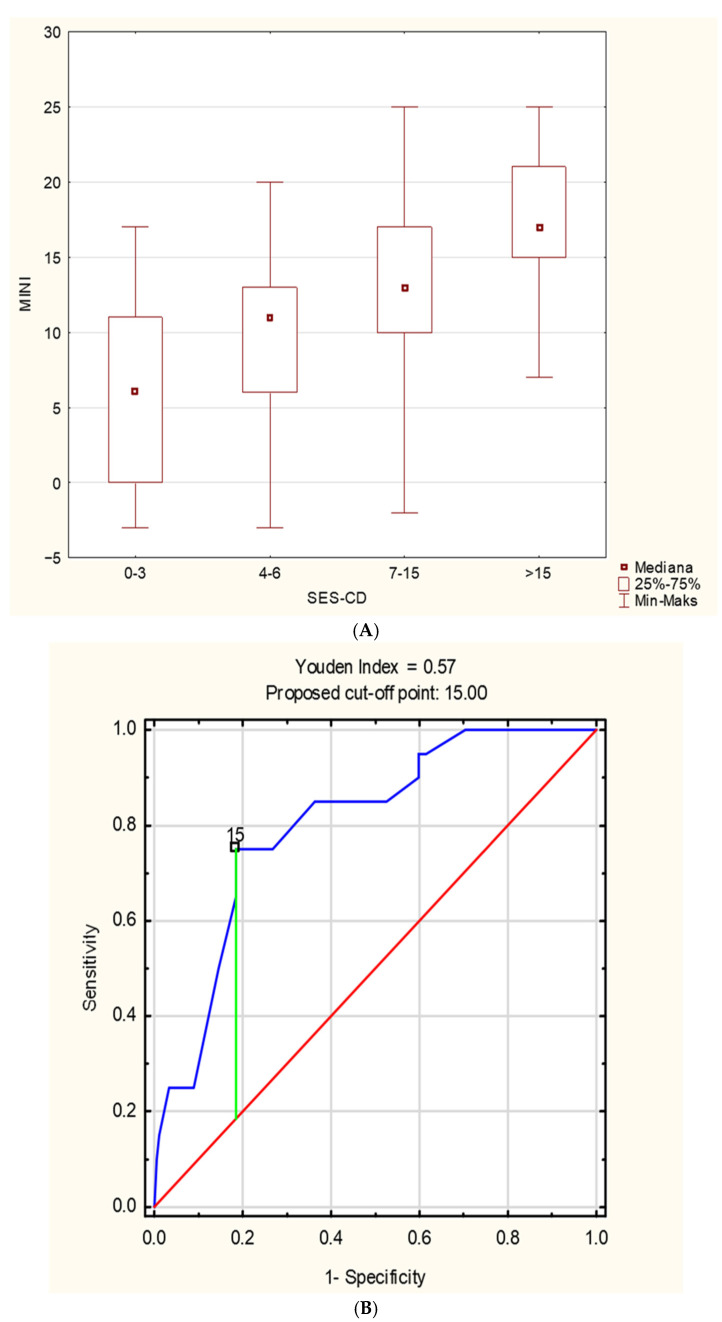

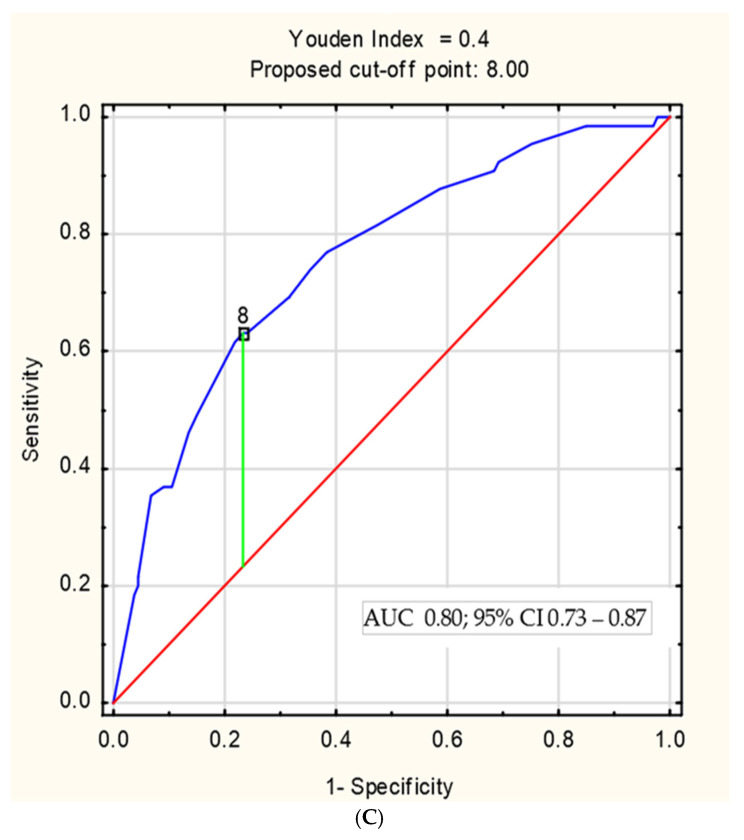
(**A**) The relationship between MINI and the severity of inflammatory changes assessed by the SES-CD. (**B**) The ROC curve of MINI in the diagnosis of severe Crohn’s disease in children, defined as SES-CD ≥ 16 points; (**C**) the ROC curve of MINI in the identification of patients with mucosal healing, defined as SES-CD < 3 points.

In the study group, a positive correlation was found between MINI and PCDAI (r = 0.52, *p* < 0.001) (Figure 1A) and between MINI and SES-CD (r = 0.54, *p* < 0.001) (Figure 1B). The relationship between MINI and the severity of inflammatory lesions assessed using the SES-CD is shown in Figure 2A. MINI score of 15 points or more indicated a severe CD (defined as SES-CD ≥ 16 points) with a diagnostic sensitivity of 86% and specificity of 89% (area under the receiver-operating characteristic curve [AUC], 0.88; 95% confidence interval [CI], 0.83–0.94). (Figure 2). Additionally, a positive correlation was demonstrated between the MINI and WBC (r = 0.39; *p* < 0.001) and PLT (r = 0.59; *p* = 0.007). The positive correlation of MINI with PLT and WBC as indirect markers of inflammation in the course of active CD additionally indicates that MINI also correlates well with other inflammatory markers than CRP, ESR, or FC.

## 5. Discussion

In this study, the correlation between MINI and currently commonly used scales of clinical and endoscopic activities (PCDAI, SES-CD) in children with CD was analyzed. This analysis showed a positive correlation between MINI and PCDAI and between MINI and SES-CD. Similar results were presented in studies by other authors, as well as in a systematic review of randomized trials [14,15,16]. In the study by Herman et al. [9] on the clinical use of MINI in children with newly diagnosed CD, MINI values higher than 17 points indicated a severe CD (defined as SES-CD ≥ 16 points) with a diagnostic sensitivity of 90% but a low specificity of 50%. In the current cohort, the cut-off point for severe CD in children was SES-CD ≥ 16 points with a similar sensitivity of 86% but with a higher specificity of 89%, similar to the study by Prokhorenkova et al. [16]. Also in the study by Prezez et al. [17], a positive correlation was shown between MINI and SES-CD (r = 0.701, *p* < 0.001).

Taking into account the results of the research conducted so far and also the results of this analysis, it can be concluded that MINI can be helpful in monitoring treatment, assessing therapeutic response, and assessing the risk of exacerbations in everyday clinical practice [6,9,18]. Herman et al. [9] point out that in children with newly diagnosed CD, endoscopy can often be difficult (e.g., lack of small bowel intubation, incomplete assessment), which increases the need for non-invasive markers. In such cases, MINI may have a role in diagnosing MH without the need for endoscopic examination of the lower GI tract. Also, during the treatment monitoring phase, there is a need to limit the number of colonoscopies to improve patients’ quality of life and reduce hospitalizations, which directly impacts public health care [6,9,18]. Currently, MINI cannot completely replace endoscopy; rather, it can complement it or be helpful in situations where endoscopy is delayed, impossible to perform, or when the FC result is intermediate [9]. Therefore, it appears that MINI may be useful for monitoring treatment, making therapeutic decisions, and monitoring the risk of relapse. However, it is important to be aware of the limitations of this index. Most studies conducted to date are single-center, characterized by small and often heterogeneous study populations and a lack of long-term follow-up, which directly impacts the generalizability of the results. Further prospective, multicenter studies are needed, involving large numbers of patients, with diverse populations, and taking into account changes over time, to determine how best to use MINI in routine pediatric care [6,9,18].

Also, this analysis has several limitations. The most significant seems to be its retrospective nature. It is a single-center study, which limits generalizability. It was conducted on a relatively small study group, without power calculations or effect size estimates, which may result in limited interpretation of statistical adequacy for the studied patient group. Another limitation is that the analysis did not address potential confounding factors, such as treatment duration or type of therapy, which could influence MINI results.

## 6. Conclusions

The results of this study indicate that MINI may be a useful tool in daily clinical practice for assessing the severity of intestinal inflammatory changes in children with CD. It should also be noted that MINI also meets other criteria for an ideal biomarker: non-invasiveness, objectivity, simplicity, and reproducibility. However, to fully understand the value of MINI in children with CD, well-designed, prospective, multicenter, and non-individual studies are necessary on a large cohort of patients, taking into account different patient groups, for example, based on age, disease location, time of diagnosis, and type of treatment.

## Figures and Tables

**Table 1:** Simple Endoscopic Score for Crohn’s Disease—SES-CD [13].

	Points			
	0	1	2	3
Size of ulcers (cm)	none	Aphtosus ulcers (diameter 0.1–0.5 cm)	Large ulcers (diameter 0.5–2 cm)	Very large ulcers (diameter > 2 cm)
Ulcerated surface (%)	none	<10%	10–30%	>30%
Affected surface (%)	unaffected	<50%	50–75%	>75%
Presence of narrowings	none	Single, can be passed	Multiple, can be passed	Can not be passed

**Table 2:** Mucosal Inflammation Noninvasive Index—MINI [6].

		Points
Stools	0–1 normal or liquid stools, no blood	0
	≤2 semi-liquid stools with a small amount of blood or 2–5 liquid stools	4
	massive bleeding or ≥6 liquid stools or nocturnal diarrhea	8
Fecal calprotectin (FC) [µg/g]	50	−3
	50–99.9	0
	100–299.9	5
	300–599.9	7
	600–899.9	9
	≥900	12
Erythrocyte sedimentation rate (ESR) [mm/h] C-reactive protein (CRP) [mg/L]	ESR < 10 and CRP < 5	0
	30 > ESR ≥ 10 or 10 > CRP ≥ 5	1
	50 > ESR ≥ 30 or 30 > CRP ≥ 10	2
	ESR ≥ 50 or 30 > CRP ≥ 30	5
Sum		−3 to 25

**Table 3:** Characteristics of the study group.

Number of Patients		199
Sex	Female	74 (37.19%)
	Male	125 (62.1%)
Age (years)	Mean	13.23
	±SD	3.34
	Range (min–max)	1–17.5
	Median	13.02
Body weight (kg)	Mean	47.29
	±SD	18.75
	Range (min–max)	8.38–113
	Median	46.6
Height (cm)	Mean	156.59
	±SD	19.77
	Range (min–max)	82–190
	Median	160
Location of the disease	L1—distal 1/3 ileum ± limited caecal disease	68 (34.17%)
	L2—colonic	82 (41.20%)
	L3—ileocolonic	49 (24.62%)
	L4—upper disease	36 (18.09%)
Disease behavior	B1—non-stricturing non-penetrating	161 (80.90%)
	B2—stricturing	37 (18.59%)
	B3—penetrating	4 (2.01%)
	B2B3—both stricturing and penetrating	3 (1.51%)
Perianal disease	p	41 (20.6%)
Growth delay	G1	31 (15.58%)

## Data Availability

Data are available from the corresponding author upon reasonable request.
